# Fear of childbirth in primipara women and its correlation with premenstrual syndrome: A cross-sectional study

**DOI:** 10.1371/journal.pone.0332135

**Published:** 2025-10-17

**Authors:** Yanran Li, Yi Lu, Xujuan Xu

**Affiliations:** 1 School of Nursing and Rehabilitation, Nantong University, Nantong, Jiangsu, China; 2 Nantong Tumor Hospital, Nantong, Jiangsu, China; 3 Affiliated Hospital of Nantong University, Nantong, Jiangsu, China; Instituto Nacional de Salud Publica, MEXICO

## Abstract

**Introduction:**

Fear of Childbirth (FOC) is the most common psychological problem among pregnant women, especially in primipara women who are more likely to fear the unknown delivery process. The causes of FOC are complex and influenced by various factors, but the impact of menstruation-related factors remains unclear. Therefore, this study aims to understand the current situation of FOC in primiparous women during late pregnancy, investigate the influencing factors of FOC, and further explore the correlation between premenstrual syndrome (PMS) and FOC.

**Methods:**

This study is a multicenter cross-sectional study. A convenience sampling method is used to select 597 primiparous women who attend regular prenatal check-ups at three healthcare institutions in Nantong City, Jiangsu Province, China, between December 2023 and February 2024. Participants are required to complete the general information questionnaire, Premenstrual Syndrome Scale, Childbirth Attitude Questionnaire, Perceived Social Support Scale, Pittsburgh Sleep Quality Index, and Marital Adjustment Scale. Mann-Whitney U test and Kruskal-Wallis test are used to access the between-group differences. Multiple linear regression is used to analyze the influencing factors of FOC. Spearman correlation examines the relationship between PMS and FOC.

**Results:**

The results show that 46.73% of primiparous women experience FOC. Multiple linear regression analysis shows that dysmenorrhea, unintended pregnancy, PMS, social support, sleep quality, and marital relationship are significant factors influencing FOC (*P* < 0.05). Additionally, PMS is moderately positively correlated with FOC (r = 0.549, *P* < 0.01).

**Conclusion:**

Our study results indicate that the social issue of FOC deserves attention. Clinical healthcare professionals should focus on the physical and mental well-being of pregnant women, particularly those with PMS. They should enhance prenatal psychological assessments and provide targeted emotional support based on individual circumstances, helping these women better cope with childbirth, improve their childbirth experience, and ensure maternal and infant health.

## Introduction

Fear of childbirth (FOC) has emerged as a significant psychological concern during pregnancy, gaining considerable attention in recent years [1]. It refers to the emotional experiences of pregnant women ranging from worry and fear to extreme anxiety, and even a desire to escape from childbirth. As the delivery date approaches, FOC tends to peak during the late pregnancy. Reports indicate that FOC affects 5% to 40% of pregnant women worldwide [2]. The etiology of this fear is complex and may be influenced by various factors. Research from Thailand has identified several key factors associated with FOC, including age, history of miscarriage, number of previous births, education level, economic status, and psychiatric history [3]. A cross-sectional study from Turkey found that low education, unemployment, low economic status, negative birth experiences, unintended pregnancies, and insufficient social support are significant risk factors contributing to FOC [4]. Previous studies have shown that FOC increases catecholamine release, intensifying delivery pain and leading to complications such as dystocia, hemorrhage, and severe perineal tears, as well as fetal distress and low birth weight in newborns [5–7]. In addition, some researchers believe that FOC may be a sign of underlying depression, as this fear is highly correlated with antenatal depression and can also predict the occurrence of postpartum depression [8,9]. Given the adverse effects of FOC on both mothers and infants, identifying the factors influencing this fear is crucial for preventing negative pregnancy outcomes, reducing the cesarean section rate, and improving the psychological well-being of pregnant women.

The menstrual period is a significant physiological phase for expectant mothers before pregnancy. Premenstrual syndrome (PMS), as one of the common gynecological diseases affecting women during the menstrual cycle, is defined in the fifth edition of the Diagnostic and Statistical Manual of Mental Disorders as a recurrent disorder characterized by a combination of psychological and physical symptoms during the luteal phase of the menstrual cycle (from ovulation to the onset of menstruation). Its main clinical manifestations include anxiety, depression, headaches, sleep disorders, abdominal bloating, diarrhea, and breast distending pain [10]. The recurrence of these symptoms may weaken women’s sense of control over their bodies and emotions, leading to low self-esteem and an increased risk of mental health disorders.

Recently, study by Hacer Ataman reported a correlation between PMS and FOC in women who have not yet been pregnant [11]. However, compared to nulliparous women, we are more eager to understand the impact of PMS on FOC in women during late pregnancy. Primipara, as a special group, often have significant concerns about the pain, complications, and uncontrollable factors associated with the delivery process due to their lack of experience. Especially as delivery approaches, they are more likely to intensify their FOC. Therefore, the factors influencing FOC in the primipara group deserve further attention.

Consequently, this study aims to investigate the current situation of FOC among primipara women in late pregnancy, understand the influencing factors, and further explore the correlation between PMS and FOC. The findings will provide scientific evidence for developing relevant interventions to alleviate FOC among pregnant women, enhance their self-efficacy during childbirth, and improve their psychological health.

## Materials and methods

### Study design and participants

This study is a multicenter cross-sectional study. A convenience sampling method is employed to select primiparous women undergoing regular prenatal check-ups at three healthcare institutions in Nantong, Jiangsu Province, China, from December 2023 to February 2024. Inclusion criteria: age ≥ 18 years; singleton pregnancy; gestational age ≥ 28 weeks; attending regular prenatal check-ups at the hospital and planning to give birth there; able to understand and independently complete the questionnaire; voluntary participation in the study. Exclusion criteria: history of mental illness; severe heart, liver, or kidney disease; pregnancy complications. According to the rough sample size estimation method proposed by Kendall, the sample size for this study is between 210 and 420 cases. Among the eligible participants, we exclude 7 women who take less than 5 minutes to complete the questionnaire and 15 women with incomplete responses. Ultimately, the study includes 597 primiparous women. The study was reviewed and approved by the Ethics Committee of Affiliated Hospital of Nantong University (approval number: 2023-K164-01). All participants voluntarily participate in the study and sign a written informed consent form, confirming that their participation is voluntary and they can withdraw from the study at any time.

### Research tools

#### General information form.

This form is designed by the researchers and includes the following information: age, residence, education level, employment status, average monthly family income, mode of medical care provider payment, pre-pregnancy body mass index, current body mass index, age at menarche, menstrual cycle, menstrual duration, dysmenorrhea, conception mode, unintended pregnancy, and history of abortion.

#### Premenstrual Syndrome Scale (PSS).

PSS is developed based on John Bancroft’s diagnostic criteria for PMS. The scale consists of 12 items, requiring respondents to indicate whether they experience related symptoms during the 14 days leading up to their last menstrual period. The total score ranges from 0 to 36, with higher scores indicating more severe symptoms. A total score greater than 6 indicates the presence of PMS [12]. The Cronbach’s α coefficient measured in this study is 0.841.

#### Childbirth Attitudes Questionnaire (CAQ).

CAQ created by Tanglakmankhong K is used to assess the level of FOC in pregnant women. It consists of four dimensions: fear of pain, fear of losing control during delivery, fear for the child’s health, and fear of the hospital environment and interventions. The total score ranges from 16 to 64, with higher scores indicating greater FOC. A total score greater than 27 indicates the presence of FOC [13]. The Cronbach’s α coefficient measured in this study is 0.774.

#### Perceived Social Support Scale (PSSS).

PSSS is designed by Zimet in 1987 to evaluate respondents’ perceptions of support from their social relationships. The scale consists of three subscales: family support, friend support, and other support. Total scores range from 12 to 84, with higher scores indicating greater perceived social support. The specific criteria are as follows: low support (12–36), moderate support (37–60), and high support (61–84) [14]. The Cronbach’s α coefficient measured in this study is 0.91.

#### Pittsburgh Sleep Quality Index (PSQI).

PSQI is a self-report questionnaire developed by Buysse in 1989 to assess sleep quality over the past month. The scale consists of seven dimensions: sleep duration, sleep quality, sleep disorders, sleep efficiency, use of sleep medications, time taken to fall asleep, and daytime dysfunction. Total scores range from 0 to 21, with lower scores indicating better sleep quality. A score greater than 7 is used as the cutoff for sleep quality issues in adults [15]. The Cronbach’s α coefficient measured in this study is 0.708.

#### Marital Adjustment and Prediction Test (MAT).

MAT is designed by Lock and Wallace in 1959 to assess the quality of marital relationships [16]. Total scores range from 2 to 158, with higher scores indicating greater intimacy in the marriage. A score of 100 or above indicates a good level of intimacy, while a score below 100 suggests a poor marital relationship. The Cronbach’s α coefficient measured in this study is 0.869.

### Statistical analysis

All data are processed using IBM SPSS 29.0 statistical software, with the following steps: non-normally distributed quantitative data are described using median and interquartile range, while categorical data are described using frequency and composition ratio. Mann-Whitney U test is used for two-sample comparisons, and Kruskal-Wallis test is used for multiple samples to assess significant differences between groups. FOC serves as the dependent variable, and factors showing statistical differences in univariate analysis undergo multiple linear regression analysis to identify their effects on FOC. The significance test level was α = 0.05, and the P value was two-sided probability. P < 0.05 indicated a statistically significant difference. Spearman correlation analysis is used to examine the relationship between PMS and FOC.

## Results

### Baseline characteristics

Among the 597 primiparous women, 26 are aged 35 or older. The majority come from urban areas, and most have a college degree or higher. Currently, 26.97% are unemployed. The average monthly family income typically ranges between 5001 and 10000 yuan, and most use medical insurance for expenses. There are 24 cases of menarche occurring before age 12 or after age 16. Additionally, 9.88% have menstrual cycles longer than 35 days, while 15 women experience menstrual periods lasting more than 7 days. Normal menstrual bleeding volume is reported by 86.26% of the women. Dysmenorrhea history is present in 43.05%. Pre-pregnancy body mass index (BMI) falls within the normal range for 57.62%, but during late pregnancy, 75.21% exceed overweight standards. Assisted reproductive technology is used by 15.08% of women, unintended pregnancy occurs in 9.55% of women, and 19.77% of women have a history of abortion (Table 1).

**Table 1:** Baseline characteristics of primiparous women.

Items	Groups	Numbers	Proportion
Age	<35	571	95.64%
	≥ 35	26	4.36%
Residence	Urban	465	77.89%
	Countryside	132	22.11%
Education level	Junior high school and below	17	2.85%
	High school/technical secondary school	48	8.04%
	College/Undergraduate	477	79.90%
	Master degree or above	55	9.21%
Employment status	Employed	436	76.72%
	Unemployed	161	23.28%
Average monthly family income (yuan)	<3000	3	0.50%
	3000-5000	107	17.92%
	5001-10000	330	55.28%
	>10000	157	26.30%
Mode of medical care provider payment	Medical insurance	499	83.58%
	Self pay	98	16.42%
Age at menarche	<12	17	2.85%
	12-16	573	95.98%
	>16	7	1.17%
Menstrual cycle (days)	21-35	538	90.12%
	>35	59	9.88%
Menstrual duration (days)	3-7	582	97.49%
	>7	15	2.51%
Menstrual bleeding volume	Low	47	7.87%
	Moderate	515	86.26%
	High	35	5.86%
Dysmenorrhea	No	340	56.95%
	Yes	257	43.05%
Pre-pregnancy body mass index	<18.5	81	13.57%
	18.5 ≤ BMI < 24	344	57.62%
	24 ≤ BMI < 28	117	19.60%
	BMI ≥ 28	55	9.21%
Current body mass index	<18.5	6	1.01%
	18.5 ≤ BMI < 24	142	23.79%
	24 ≤ BMI < 28	230	38.53%
	BMI ≥ 28	219	36.68%
Conception mode	Natural	507	84.92%
	Assisted reproductive technology	90	15.08%
Unintended pregnancy	Yes	97	9.55%
	No	500	90.45%
History of abortion	No	479	80.23%
	Yes	118	19.77%

### Scores of various scales

Among the 597 primipara women, 27.14% reported having PMS, 46.73% experienced FOC, 0.34% reported low social support, 17.09% reported moderate social support, and 82.58% reported high social support. Additionally, 26.47% of the pregnant women experience sleep disorders, and 8.21% reported poor marital relationships (Table 2).

**Table 2:** Scores of various scales in primiparous women.

Items	Groups	Numbers	Proportion
PMS	No (PMS < 6)	435	72.86%
	Yes (PMS ≥ 6)	162	27.14%
FOC	No (CAQ ≤ 27)	318	53.27%
	Yes (CAQ > 27)	279	46.73%
Social support	Low (12 ≤ PSSS ≤ 36)	2	0.34%
	Moderate (37 ≤ PSSS ≤ 60)	102	17.09%
	High (61 ≤ PSSS ≤ 84)	493	82.58%
Sleep quality	Good (PSQI ≤ 7)	439	73.53%
	Poor (PSQI >7)	158	26.47%
Marital relationship	Low (MAT < 100)	49	8.21%
	High (MAT ≥ 100)	548	91.79%

### Univariate analysis of FOC

In the univariate analysis of FOC, factors such as employment status, mode of medical care provider payment, menstrual duration, dysmenorrhea, unintended pregnancy, PMS, social support, sleep quality, and marital relationship show significant statistical differences (*P* < 0.05) (Table 3).

**Table 3:** Univariate analysis of FOC in primiparous women.

Items	Groups	FOC [M (P25, P75)]	Z/H	*P*
Age	<35	[27 (21, 33)]	−0.376	0.707
	≥ 35	[26 (22, 32.5)]		
Residence	Urban	[26 (21, 32)]	−0.746	0.456
	Countryside	[28 (20, 35)]		
Education level	Junior high school and below	[28 (19, 38.5)]	3.133	0.372
	High school/technical secondary school	[27 (20, 34.5)]		
	College/Undergraduate	[26 (21, 32)]		
	Master degree or above	[29 (23, 35)]		
Employment status	Employed	[25 (20, 31)]	−5.903	<0.001*
	Unemployed	[31 (25, 36.5)]		
Average monthly family income (yuan)	<3000	[26 (22.5, 26.5)]	5.063	0.167
	3000-5000	[24 (19, 32)]		
	5001-10000	[27 (22, 33)]		
	>10000	[27 (21, 32)]		
Mode of medical care provider payment	Medical insurance	[26 (21, 32)]	−2.742	0.006*
	Self pay	[30 (22, 36.25)]		
Age at menarche	<12	[26 (22.5, 30.5)]	0.226	0.893
	12-16	[27 (21, 33)]		
	>16	[28 (20, 29)]		
Menstrual cycle (days)	21-35	[27 (21, 32)]	−0.258	0.797
	>35	[26 (20, 34)]		
Menstrual duration (days)	3-7	[26 (21, 32)]	−2.686	0.007*
	>7	[33 (26, 40)]		
Menstrual bleeding volume	Low	[30 (20, 39)]	4.031	0.133
	Moderate	[26 (21, 32)]		
	High	[27 (22, 32)]		
Dysmenorrhea	No	[23 (19, 30)]	−7.542	<0.001**
	Yes	[29 (24.5, 37)]		
Pre-pregnancy body mass index	<18.5	[28 (21, 35)]	4.883	0.181
	18.5 ≤ BMI < 24	[27 (21, 32.75)]		
	24 ≤ BMI < 28	[26 (20, 32)]		
	BMI ≥ 28	[25 (19, 30)]		
Current body mass index	<18.5	[24 (17, 35.25)]	4.021	0.259
	18.5 ≤ BMI < 24	[28 (22, 35)]		
	24 ≤ BMI < 28	[26 (21, 32)]		
	BMI ≥ 28	[26 (20, 32)]		
Conception mode	Natural	[27 (21, 32)]	−0.323	0.747
	Assisted reproductive technology	[26 (20, 33)]		
Unintended pregnancy	No	[25.5 (20, 31)]	−6.124	<0.001**
	Yes	[32 (26, 39.5)]		
History of abortion	No	[26 (21, 33)]	−0.919	0.358
	Yes	[28 (21, 32)]		
Premenstrual sydrome	No	[24 (19, 30)]	−9.274	<0.001**
	Yes	[31.5 (28, 38.25)]		
Social support	Low	[49 (37, 61)]	33.027	<0.001**
	Moderate	[31 (24, 39)]		
	High	[26 (20, 31)]		
Sleep disorders	No	[25 (20, 30)]	−8.133	<0.001**
	Yes	[32 (27, 39)]		
Marital relationship	Low	[33 (28, 40)]	−4.712	<0.001**
	High	[26 (20, 32)]		

*: *P* < 0.05, **: *P* < 0.001.

### Multivariate linear regression analysis of factors influencing FOC

Use FOC as the dependent variable and include the statistically significant independent variables from the univariate analysis in the multiple linear regression model. The VIF values for the variables in the model range from 0.712 to 0.972, and the tolerance values range from 1.029 to 1.404, indicating no multicollinearity among the variables. From the standardized coefficients β, dysmenorrhea, unintended pregnancy, PMS, social support, sleep quality, and marital relationship are significant factors affecting FOC (**P* *< 0.05) (Table 4).

**Table 4:** Multivariate linear regression analysis of factors influencing FOC in primiparous women.

	Unstandardized coefficient		Standardized coefficient	t	*P*	Collinearity Statistics	
	B	Standard error	Beta			Tolerance	VIF
Constant	32.068	3.267		9.815	<0.001		
Employment status	1.464	0.735	0.072	1.993	0.047*	0.812	1.232
Mode of medical care provider payment	0.17	0.848	0.007	0.201	0.841	0.875	1.143
Menstrual duration	2.997	1.904	0.052	1.574	0.116	0.972	1.029
Dysmenorrhea	2.576	0.642	0.141	4.012	<0.001**	0.854	1.171
Unintended pregnancy	3.067	0.844	0.125	3.633	<0.001**	0.889	1.124
PMS	0.74	0.109	0.261	6.796	<0.001**	0.712	1.404
Social support	−0.116	0.031	−0.137	−3.768	<0.001**	0.792	1.262
Sleep quality	0.591	0.111	0.194	5.32	<0.001**	0.789	1.267
Marital relationship	−0.041	0.016	−0.091	−2.514	0.012*	0.802	1.247

*: *P* < 0.05, **: *P* < 0.001.

### Spearman correlation analysis of PMS and FOC

Spearman correlation analysis shows a moderate positive correlation between PSS and both the total score and individual dimension scores of CAQ (r = 0.446–0.549, **P* *< 0.01) (Table 5).

**Table 5:** Spearman correlation analysis of PMS and FOC in primiparous women.

Variables	PSS	CAQ	F1	F2	F3	F4
PSS	1					
CAQ	0.549**	1				
F1	0.523**	0.890**	1			
F2	0.446**	0.682**	0.628**	1		
F3	0.532**	0.898**	0.802**	0.609**	1	
F4	0.533**	0.885**	0.815**	0.647**	0.789**	1

**: *P* < 0.01.

F1: Fear of Losing Control during Delivery.

F2: Fear of the Hospital Environment and Interventions.

F3: Fear for the Child’s Health.

F4: Fear of Pain.

## Discussion

The prevalence of FOC varies significantly between countries. In developed nations such as the United States, Canada, and Australia, the rates are relatively low, typically ranging from 6.3% to 14.8% [17]. In contrast, developing countries report much higher prevalence rates. For example, in Vietnam, 30.91% of pregnant women experience FOC [18], while in Turkey, the rate is 42.2% [4], and in Egypt, it also reaches 42.2% [19]. These developing countries may face challenges such as limited medical resources, inadequate prenatal education, and weak social support systems, which contribute to heightened FOC among women. In China, the situation is similar to that of other developing nations, with reports from Chongqing indicating a fear prevalence of 67.1% [13], and even higher rates of 70.3% among healthy pregnant women in the northwest region [20]. Our study finds that 46.73% of primiparous women experience FOC, underscoring the need for greater attention to this issue within this population.

Our study explores several factors significantly influencing FOC among primiparous women. The results indicate that sleep quality is a key factor affecting this fear, aligning with findings from research in South China [21]. Good sleep quality helps the brain manage emotions and stress, while poor sleep diminishes emotional regulation, making women more prone to worries and anxiety about childbirth. Additionally, our study confirms the major impact of marital relationships and social support on FOC, consistent with the conclusions of A. Ezgi Ulu and Aysu BULDUM [22,23]. Insufficient social support during pregnancy from spouses, parents, or friends exacerbates psychological stress, increasing fear due to a lack of emotional backing. We also find that unintended pregnancy contributes to heightened FOC, a conclusion supported by research in Saudi Arabia [24]. Compared to planned pregnancies, unplanned ones often leave women psychologically unprepared for childbirth, leading to increased anxiety and fear about the future, further intensifying their FOC.

Our findings not only align with previous research but also reveal the potential impact of menstrual-related factors on FOC. Hacer Ataman and colleagues identified a correlation between PMS and pre-pregnancy FOC among non-pregnant women [11]. Our study further confirms the influence of PMS on FOC in late pregnancy. As a recurring condition in the menstrual cycle, PMS can be seen as a cyclical source of psychological and physiological stress. Women with PMS often experience emotional fluctuations, which can lead to chronic stress, making them more susceptible to excessive fear when facing high-pressure events like childbirth [25]. Additionally, dysmenorrhea, a symptom of PMS, is also an influencing factor in our research. Women who experience dysmenorrhea carry vivid memories of pain, making them more likely to associate childbirth with intense pain. This long-term pain memory heightens sensitivity to pain and reduces pain tolerance, amplifying their fear of labor pains and resulting in an increased FOC [26].

To our knowledge, our study is the first to analyze the influence of menstrual-related factors and PMS on FOC, confirming the potential impact of women’s physiological cycles on their psychological state during pregnancy. Additionally, while previous studies have largely explored the factors influencing FOC in the general pregnant population, this study focuses on the specific group of primiparas to further analyze the factors affecting their FOC. Finally, this study collects a relatively large sample size through a multi-center cross-sectional survey, covering primiparous women from different medical institutions, which may to some extent reflect the psychological characteristics of the population in a single region of China. However, future research still needs to investigate more diverse populations across different countries and regions to further validate the generalizability of the findings.

However, our study has certain limitations. Firstly, the data in this study mainly come from self-reports by pregnant women, which may introduce subjective bias. Specifically, the assessment of PMS uses the PSS, a retrospective tool that may overestimate the prevalence of PMS due to recall bias. While recall bias affects the reporting of PMS symptoms, it is important to note that PMS is a cyclical condition, and women experience these physical and emotional symptoms repeatedly over the years, leading to a certain stability in their individual symptoms. Furthermore, for pregnant women in late pregnancy, their last menstrual period may have occurred only a few months prior, resulting in a relatively short time span. This is particularly true for those with pronounced PMS symptoms, who are likely able to recall their past symptoms accurately. Additionally, since our study employs a cross-sectional design, it cannot establish causal relationships between the various factors and FOC. Finally, this study may still have unaddressed confounding factors. For example, one limitation is the exclusion of pregnant women’s anticipated delivery mode (e.g., planned vaginal delivery versus elective cesarean section) as a primary variable in the analysis. Although the anticipated delivery mode may influence the severity of FOC, China’s current maternal delivery policies encourage healthy pregnant women to prioritize vaginal delivery in the absence of medical indications for cesarean section, which results in fewer cases of elective cesarean sections. This policy context limits our ability to thoroughly explore this variable in the study. Furthermore, the psychological state during pregnancy is an important factor that influences FOC, but this study does not comprehensively measure or control for levels of anxiety and depression during pregnancy. Future studies can incorporate standardized psychological assessment scales in their design to further control for the potential impact of emotional variables on FOC, thereby more accurately elucidating the independent association between PMS and FOC.

While our study explores the impact of PMS on FOC, the physiological and psychological mechanisms underlying this relationship remain unclear. Future research investigates whether PMS influences the occurrence of FOC through mediating factors such as psychological resilience, pain perception, and coping strategies. Additionally, prospective cohort studies further validate the long-term association between PMS and FOC by tracking women planning for pregnancy, observing how the persistence of PMS symptoms affects their childbirth experiences and mental health during pregnancy and postpartum. Currently, psychological intervention studies addressing the relationship between PMS and FOC are scarce. Future efforts explore how interventions targeting PMS can effectively reduce the risk of developing FOC and assess the efficacy of these intervention strategies.

This study highlights the importance of healthcare providers addressing women’s mental health and physiological symptoms during prenatal care. It is recommended that healthcare professionals incorporate screening for PMS symptoms into prenatal assessments to understand the physiological and psychological states of expectant mothers. Firstly, it is recommended that healthcare institutions provide training for obstetricians and nurses on PMS. The training should cover the identification of PMS symptoms, the potential association with FOC, and the use of screening tools. The training can be conducted through online courses or workshops to ensure that healthcare professionals are proficient in assessing PMS symptoms and identifying mental health risks in pregnant women. Secondly, PMS screening should be conducted during the initial prenatal visit. When registering pregnant women, obstetricians should use a standardized PMS scale combined with clinical interviews to assess whether the woman has experienced significant PMS symptoms in the past, identifying high-risk individuals. Healthcare professionals should record PMS screening results in the prenatal care manual and monitor psychological changes throughout pregnancy. For those identified with significant PMS symptoms, personalized psychological support measures such as cognitive behavioral therapy, mindfulness training, or emotional support interviews should be offered to help boost their confidence in coping with childbirth and reduce FOC. In addition, healthcare providers should incorporate a psychological health module on PMS and FOC in prenatal education. Psychologists or obstetric experts should be invited to give lectures to pregnant women and their families, explaining how PMS symptoms may impact the childbirth experience. This can help pregnant women better understand their emotional responses and enhance their psychological adjustment abilities. Finally, healthcare institutions can establish a continuous mental health record system, tracking women with a history of PMS from pre-pregnancy to postpartum. This allows for ongoing follow-up to observe psychological changes and prevent the development of more severe mental health issues such as postpartum depression. In addition to screening and intervention, healthcare professionals should focus on communication with pregnant women and build a supportive network. They should care for their sleep quality during pregnancy and encourage family members and friends to listen to their concerns about childbirth. Overall, this research provides critical insights for clinicians in identifying risk factors associated with FOC. In future prenatal care, emphasis should be placed on early screening of high-risk groups for fear of childbirth. Developing systematic and individualized psychological intervention plans, along with strengthening prenatal education related to childbirth, will help expectant mothers establish a solid social support system. This approach effectively reduces maternal fear of childbirth and promotes maternal and infant health.

## Conclusion

Our study results indicate a high prevalence of FOC among primiparous women. Clinical healthcare providers should early identify risk factors associated with FOC, particularly focusing on screening for PMS. Enhancing prenatal care and strengthening social support systems are essential to alleviate FOC and ensure the health of mothers and infants.

## Supporting information

S1 Dataset Data file for open access. (XLSX)
